# Observations on Dr. Scudamore's Reply to Mr. Bampfield's Paper on the Use of Colchicum in Gout; with a Vindication of Himself, and a Farther Defenoe of the Character and Utility of That Medicine in the Treatment of That Disease

**Published:** 1822-02

**Authors:** R. W. Bampfield

**Affiliations:** one of the Surgeons to the Royal Metropolitan Infirmary for Sick Children, &c. &c.


**Art. II.—*Observations on* Dr. Scudamore's *Reply to* Mr. Bampfield's *Paper on the Use of Colchicum in Gout; with a Vindication of himself, and a farther Defence of the Character and Utility of that Medicine in the Treatment of that Disease.* By R. W. Bampfield, Esq. one of the Surgeons to the Royal Metropolitan Infirmary for Sick Children, &c. &c.**

---

"I will not apologize for the freedom which I use in criticising the statements of this distinguished author; being well assured, that, from his zeal in science, he will court the contest of opinion, where any important truth is to be established."—*Note on* Sir E. Home, *vide* Scudamore *on Gout*, 3d ed. p. 200.

---

If there be any occasion on which the public would tolerate and excuse the author of a paper from obtruding a rejoinder on their patience, that surely is one, in which he is called upon to vindicate his reputation from the gross imputation of mis-statements. Our intellectual eye perceives no difference in the turpitude of a moral or literary falsity: the latter is, perhaps, the more deliberate, and thence more inexcusable; yet, if I may be permitted to know aught of the moral construction of my own mind, it is as incapable of being wilfully guilty of "the mis-statements and misrepresentations," "*quotations of a false stamp*," "garbled and pretended quotations," with which I am charged, both in the alpha and omega of Dr. Scudamore's reply—as he ought to have been of imputing such serious dereliction of virtuous principle, without adducing undeniable proofs of it. Personal hostility to Dr. S. I disclaim; and, in the further critical freedoms I may indulge on parts of his treatise, no offence is intended; for I am actuated by a persuasion, that the good of the public, and the interests of the profession, will be promoted, and medical science advanced, if, by the pending discussion, the precise effects of colchicum in gout be indisputably ascertained, and the exact quantities necessary to produce those effects be clearly established. Thus we may hope to dispel those discrepancies of opinion existing on the subject, and reconcile those differences of practical results that have occurred to Dr. S. and myself, which, else, would have appeared almost irreconcileable, had not the concessions already made by Dr. S. induced me to think the desideratum within our power.

With the respect that is due to truth, I frankly confess that I have unconsciously misquoted the Pharmacopœia Lond.; and Dr. S. is fairly entitled to all the advantages arising from this oversight, and *all* the incorrectness to which that mistake has led: but we fearlessly challenge him to prove that we have, in one single instance, ever misquoted his treatise, or misrepresented or mis-stated the quoted passages, or have attempted to pervert his meaning from its just and obvious sense. By placing my quotations in juxta-position with the passages in his treatise, the public will be enabled to form their judgment; and, as Dr. S. thinks it would have been a more "regular proceeding to have searched for his sentiments in the third (or last) edition," I have again collated the passages in the second and third edition, and find them *verbatim* the same; as the reader may satisfy himself, by turning to the references.

| *The passages quoted from the second edition of Dr. S.'s Treatise.* | *The same quoted from the third, or last, edition of Dr. S.'s Treatise.* | *The same, as they are quoted by Mr. B.* |
|---|---|---|
| "I have repeatedly also made trial both of the powder and the tincture,* but *from neither have I been able to trace the smallest specific operation.* It was taken in free doses; *but with effects altogether unsatisfactory,* when used in either of these forms, and relied upon as the only medicine. I observed that the stomach was irritated; an increased fur of the tongue, with thirst, were produced; and no certain action of the bowels occurred."—*2d ed.* p. 193. | "I have repeatedly also made trial both of the powder and the tincture, but from neither have I been able to trace the smallest specific operation. It was taken in free doses; but with effects altogether unsatisfactory, when used in either of these forms, and relied upon as the only medicine. I observed that the stomach was irritated; an increased fur of the tongue, with thirst, were produced; and no certain action of the bowels occurred."—*3d ed.* p. 194. | "Dr. S. denies that colchicum possesses either 'specific virtues† or operation in gout.'—P. 193, *2d ed.* "Dr. S. in speaking more particularly of this medicine, given 'in free doses,' says, 'its effects were altogether unsatisfactory;' and he 'observed that the stomach was irritated; an increased fur of the tongue, with thirst, were produced; and no certain action of the bowels occurred.'—P. 193, *2d ed.* |
| "The colchicum, brought to the state of a mere solution in water, by having its acid menstruum neutralized, is in the most favourable state of preparation in which it can possibly be administered. A very small portion of magnesia proves sufficient to neutralize the acetic acid."—*Note*, p. 186, *2d ed.* | "The colchicum, brought to the state of a mere solution in water, by having its acid menstruum neutralized, is in the most favourable state of preparation in which it can possibly be administered. A very small portion of magnesia proves sufficient to neutralize the acetic acid."—*Note*, p. 186, *3d ed.* | "In the formula alluded to, the acetic acid is neutralized by magnesia, and 'the colchicum, brought to the state of a mere solution in water, by having its acid menstruum neutralized, is in the most favourable state of preparation in which it can possibly be administered.' |
| "I have experienced the most remarkable success from a draught composed of magnesia, gr. xv. ad xx.; magnes. sulphat. ʒj. ad ʒij.; aceti colchici, ʒj. ad ʒij.; with any distilled water the most agreeable, and sweetened with any pleasant syrup, or with 15 or | "I have experienced the most remarkable success from a draught composed of magnesia, gr. xv. ad xx.; magnes. sulphat. ʒj. ad ʒij.; aceti colchici, ʒj. ad ʒij.; with any distilled water the most agreeable, and sweetened with any pleasant syrup, or with 15 or | "I have experienced the most remarkable success from a draught of magnesia, gr. xv. ad xx.; magn. sulphat. ʒj. ad ℥ij.; aceti colch. ʒj. ad ʒij.; with any distilled water the most agreeable, and sweetened with syrup or extr. glycyrrhiz. |

* The Tr. and wine are generally linked by the conjunction copulative, *and*, in the list of pretended specifics by Dr. S.—*See p.* 196 *and* 197 *of his Treatise.*

† For authority in using the word "virtues," see p. 193, Dr. S.'s 2d ed.

| *Passages quoted from the second Edition.* | *The same from the third Edition.* | *Mr. B.'s quotations.* |
|---|---|---|
| 20 grains of extr. glycyrrhiz."—p. 185-6, 2*d ed.* | 20 grains of extr. glycyrrhiz."—*See* p. 185-6, 3*d ed.* | |
| "It should be repeated at intervals of four, six, or eight, hours."—P. 186, 2*d ed.* | "It should be repeated at intervals of four, six, or eight, hours."—P. 186, 3*d ed.* | "It should be repeated at intervals of four, six, or eight, hours." |
| "The proximate cause of a disease must not only be something which is invariably antecedent, but also that which is distinctly peculiar to such disease."—P. 130, 2*d ed.* | "The proximate cause of a disease must not only be something which is invariably antecedent, but also that which is distinctly peculiar to such disease."—P. 130, 3*d ed.* | "The proximate cause of a disease must not only be something which is *invariably antecedent*, but that which is distinctly peculiar."—*M. and P. Jour., Nov.* 1821, p. 445. |
| "We are now brought to the general conclusion, that gout is a disease depending upon a redundancy of blood, with relation to the powers of the circulation, particularly affecting the system of the vena portarum, and the consequent functions of the liver; together with the production of a morbid change in the secreted products of the alimentary canal in general, and of the kidneys in particular."—P. 141-2, 2*d ed.* | "We are now brought to the general conclusion, that gout is a disease depending upon a redundancy of blood, with relation to the powers of the circulation, particularly affecting the system of the vena portarum, and the consequent functions of the liver; together with the production of a morbid change in the secreted products of the alimentary canal in general, and of the kidneys in particular."—P. 141-2, 3*d ed.* | "We are now brought to the general conclusion, that gout is a disease depending upon a redundancy of blood, with relation to the powers of the circulation, particularly affecting the system of the vena portarum, and the consequent functions of the liver; together with the production of a morbid change in the secreted products of the alimentary canal in general, and of the kidneys in particular."—*Id.* p. 446. |

It did not appear absolutely necessary to notice the two last quotations, relative to the pathology of gout; because, although Dr. S., in the first paragraph of his reply, has observed, that "it is respecting the doctrines, of which he has been an advocate, that he thinks it necessary to take up his pen on this occasion," and subsequently promises to defend his "*principia*," yet he has, unaccountably, forgotten to notice them at all, and has left the gastro-hepatic pathology, like "the blood of Douglass," to defend itself.

There is, also, in my paper, a verbatim quotation from the second edition of Dr. S.'s treatise, relative to the acetic acid taking up all the active properties of colchicum, and producing all its good effects, without being chargeable with any one ill consequence; which is entirely omitted in the last edition.—Has Dr. S. had reason to alter his opinion of the superiority of the acetum colchici to all "its other forms?"

When accusations are diffusely dealt in, the accuser should feel assured of his own correctness: in endeavouring to drive me on Scylla, Dr. S. appears to have himself fallen on Charybdis. In his reply,* it is stated to be "Mr. B.'s object to show that Dr. S. directs, in his prescriptions, *much larger* doses of colchicum than Mr. B. employs." What I have actually written is, that "it will be another object to demonstrate that Dr. S. administers *as much* colchicum as *any other* practitioner. Surely there is some striking difference between "*as much*" and "much larger," and between "Mr. B." and "any other!"† Again, he observes, Mr. B. "reckons that Dr. S., in his ordinary treatment, prescribes *four times* more of colchicum, to be taken in the space of twenty-four hours, than Mr. B. administers." The passage intended to be quoted from my paper is in these words:—"Hence, in the relative quantities given by Dr. S. and myself, in twenty-four hours, his exceeds mine *by exactly one-fourth, or twenty-five per cent.*"‡ I will not be so rude as to charge Dr. S. with a wilful mis-statement; but surely his friends cannot compliment the arithmetical accuracy of one, who has mistaken the fractional part of an unit ($\frac{1}{4}$,) for the whole number of 4, even when the decimal fraction 0.25 was added, to render the calculation clear, and to free it from all ambiguity!

"In the formula of the draught, (p. 173,)" says Dr. S. "I express one drachm as the dose."—On referring to this page, and also to 163, 183, and 193, we do not find any such passage, or discover one single word relative to the draught, or its drachm dose; so that Dr. S. here misquotes himself! "I have not, in any part of my book," says Dr. S. "*hinted* at the employment of an ounce and a half (of acetum colchici,) in the twenty-four hours; and, in point of fact, I have never given a larger quantity than six drachms." At p. 186 of both second and third (or last) edition, Dr. S. has expressly stated, that from one to *two* drachms of acetum colchici "*should be* repeated at intervals of *four*, six, or eight, hours;" and "dwells upon the importance of adapting *the activity* of this part of the treatment entirely to the degree of gouty inflammation which is present." And, to remove all doubt on the subject of the quantity to be given, Dr. S. has subsequently observed, at p. 200, (second edition,) "*I have given the draught in question, containing from one to* TWO *drachms of the acetic preparation*

---

* See *Lond. Med. and Phys. Journal*, for December 1821, p. 553.

† In another paragraph, I proceed to demonstrate, Dr. S. gives larger doses of colchicum than the generality of practitioners, who prefer the wine. Thus, perhaps, by taking part of the words "as much" in one paragraph, and "larger" in another, Dr. S. may explain his quotation.

‡ Note, p. 8.—This should have been 33$\frac{1}{3}$, or 0.333, per cent.

(*of colchicum*,) in the height of the paroxysm, *every four* or six *hours*." Now, if *two drachms* be given *every four hours*, two drachms must be repeated six times in the four and twenty, and twelve drachms, or one ounce and a half, must be administered within that period; for, 24 divided by 4 gives the quotient of 6, and 6 multiplied by 2 gives the product of 12 [drachms, equal to 1½ oz.] —4) 24 (6 × 2 = 12ʒ = 1½ ℥.

We are, however, indebted to Dr. S. for his subsequent candid confession, as it confirms our statement, that gout can be cured by smaller doses of colchicum than is generally known to the profession; and, if the third edition be prophetically termed the *last*, he must feel under an obligation to us for finding him an opportunity of correcting an error, of no ordinary moment, which has escaped his observation in the three editions of his work; and the public now understand, that, instead of the doctor having "given" from one to *two* drachms every four or six hours, as above plainly expressed, he recollects he has never given more than one drachm every four or six hours; and that the two drachms should be every where erased from his treatise, in all its editions!

I presume, Dr. S. by this time perceives that, of all the beings in the endless scale of creation, man, insignificant, little man, is the most prone to error, and should be the most considerate and lenient to the faults of his fellow-creatures: it is also certain, he may be sometimes inconsistent, and a little "at variance" with himself; for, in parag. 2d of Dr. S. reply, he loudly complains that I have not searched for his sentiments in the third edition; yet, in the *next* paragraph, he quotes, as a context, in explanation of those sentiments, a passage from the second edition, which he has wholly omitted and rejected in the third (or last) edition! This appears, to me, to be not a slight violation of consistency.

I shall proceed to state the differences in the practical results that have occurred to Dr. S. and myself, from the use of colchicum in gout; which, I hope, will prove more interesting and useful than my vindication, and the critical *exposé* of his errors.

Under the head of "pretended specifics" for gout, Dr. S. allows mercury for the cure of syphilis, and peruvian bark for the cure of intermittents, to have "a fair claim to the title of specifics." "This, however," he observes, "cannot be said of the eau medicinale; tincture of colchicum, and the vinous infusion," &c.* . . . (see the quotation below:) whilst we venture

---

* "The very general success of mercury in destroying the syphilitic virus in the system, gives it a real claim to the title of specific. The Peruvian bark deserves almost the same general praise, for its speedy and permanent control over a reto consider colchicum "a specific," or a medicine peculiarly adapted to cure gout. The results of Dr. S.'s mode of treatment of gout, by the employment of the vinous infusion and tincture of colchicum, induce him to charge them with "leading to the still more calamitous, because *more constant*, sufferings of the *chronic form of disease*."* We assure him, that, under our mode of exhibiting the vinum colchici, such a consequence has not occurred in our practice; but that, on the contrary, we have, invariably, found it to prevent the chronic form, and to cure it when present.

From Dr. S.'s mode of exhibiting those preparations of colchicum, he has observed, in a sort of tirade against the vinous infusion, that, "where it has most succeeded, relapses have so much occurred, that the disease has gained in frequency what it has lost in force."† He further asks, in his reply to my observations, if the free use of colchicum, *per se, induce, in a remarkable manner, a much greater frequency in the return of the paroxysm*, how can it be entitled to the praise of a specific cure for gout?‡ I confidently reply, that my experience in the mode of treatment I have followed does not supply a single instance of the cure by vinum colchici, *per se*, inducing a more frequent return of the paroxysm. As I have stated the precise gular intermittent fever. This, however, cannot be said of the eau medicinale; tincture of colchicum and the vinous infusion; the preparation of hellebore and opium; elaterium and opium; Wilson's tincture; and Reynolds' specific. They do, in most instances, for a few trials, influence the local symptoms very speedily; but, so far from removing the cause of gout, they leave the disposition to the disease much stronger in the system, with less powers, it is true, to produce violent inflammatory attacks; and lead to the still more calamitous, because more constant, sufferings of the chronic form of the disease. The patient emphatically describes, that his feelings make him in constant dread of something worse occurring than the gout, which his constitution no longer seems able fairly to produce. With the effects of elaterium and opium, I am the least acquainted; but I have had abundant opportunity to know that each of the other medicines, sooner or later, disappoints the patient of his expected cure, rendering merely a palliative assistance, and keeping the disease dormant for a time only, so that it is left to prey on the constitution with more lasting and serious ill effects."—Dr. SCUDAMORE's *Treatise on Gout*, p. 197-8, 3d edition.

* See the above quotation.

† "I have also witnessed the effects of the infusion recommended by Sir E. Home, and am persuaded that it is a preparation much to be preferred to the spirituous tincture. It has afforded palliative relief to many persons, for a time, to their satisfaction; to a few, more permanently; but I have been consulted by some, who have found all the ill consequences to the stomach from this infusion, which I have ascribed to the tincture, without any satisfactory influence being produced over the severe symptoms of the paroxysm. Others have derived immediate relief, but the gout has recurred much more quickly than before. With some, the relapse has taken place almost immediately, and the repetition of the medicine has appeared of no satisfactory avail. Where this remedy has most succeeded, I have observed that relapses have so much occurred, that the disease has gained in frequency what it has lost in force."—Dr. SCUDAMORE's *Treatise on Gout*, p. 199, 3d ed.

‡ The *London Med. and Phys. Journal*, for December 1821.

quantities of vinum colchici I have prescribed, and the mode adopted by me in carrying off the paroxysm, it seems incumbent on Dr. S., and he is called upon, in the name of his patronizing public, to inform them, what have been the quantities he employed, and the plan he has pursued, to produce such opposite results; for his treatise does not acquaint us with those particulars.

The great practical questions on which the doctor and I differ are these:—1st. Whether the cure of the paroxysm of gout by the vin. colchici leads to the chronic form of the disease? 2dly. Whether it induces a much greater frequency in the return of the paroxysm? 3dly. Whether colchicum be entitled to the claim of a specific remedy, or not? And on these topics we shall enter into a brief discussion, and make a few observations.

When a disease has its acute and chronic stages, experience has taught us, that the chronic usually succeeds to a severe or long acute stage; and that the chronic form is prevented when the acute attack is speedily cured. We confess, therefore, we, *primâ facie*, viewed it as a paradox, that a medicine should be charged with producing the chronic form of disease, which, in the several trials we had made of it, completely cured its acute stage within a week, and thereby prevented its chronic form: besides, it is evidently contrary to all analogy and experience, in other diseases which have a chronic sequela, that their chronic form should be the result of the speedy and effectual cure of the acute stage. Again: where we have met with the chronic modification of gout, we have been equally successful in subduing the existing gouty pain and inflammation within seven days, and have thereby cleared away the impediments to the employment of such means as are necessary to cure the lamentable consequences of chronic gout. These consequences are, ordinarily, thickened ligaments and enlarged bursæ mucosæ, producing swelled joints, thickening of the sheaths of tendons, and enlargement of the tendons, with partial adhesions between them; thickened fascia, œdema of the extremities, lameness, and chalkstones. These are all prevented by the early cure of the paroxysm; and, when produced by chronic gout, can be commonly cured (except chalkstones,) by local warm and tepid bathing,—long-continued friction, first without liniment, then with lin. camphoræ,—and pressure, by a well-applied bandage or adhesive straps.

On the first perusal of Dr. Scudamore's treatise, we thought it, at least, a feasible position, that the speedy cure of gout might lead to a more frequent return of the paroxysm; yet no other reason suggested itself to us for supporting the position, or admitting its probability, than this,—that the patient might be prompted to indulge, less scrupulously, in the luxuries of the table, or be less discreet and diligent in avoiding other exciting causes, when he had discovered he could obtain a quick and easy cure of a paroxysm of gout. Experience has proved this reason to be unsupported by facts; and we leave the speculative to explain or unfold the knowledge of the powers by which the constitution so frequently regulates the periods of the return of gout, with so much unvarying precision and surprising regularity. Patients are found subject to invasions of gout once in triennial, biennial, or annual periods, or twice, thrice, or four times a-year; and the uniformity of return is seldom interrupted in them, except by some casual and powerfully-exciting cause, that hastens the period of attack, or by some strict change of regimen, that delays it. Here, too, analogy and reason are opposed to the conclusion, that a disease recurs more frequently because it is promptly cured; for practical observation assures the profession, that, the longer a disease has continued to occupy a particular part of the body, the stronger the predisposition to future attacks has become, and *vice versâ.*

Sir H. P—, æt. 66, had been subject, for some years, to annual attacks of severe paroxysms of gout, which gradually merged into the chronic stage, and afflicted him, from two to six months, with such painful sufferings as the gouty only can comprehend. In the last attack, (January 16th, 1821,) I prevailed on him to take the vin. colchici, in doses of from twenty to thirty minims every night, and gradually carried off the pain, inflammation, and fever, in five days. The right wrist, hand, and fingers remained enlarged and œdematous; but the swelling subsided, by the employment of friction, and the application of a bandage, in ten days. In this case, the chronic stage of gout was prevented, and the frequency of recurrence has not, so far, increased, by prescribing the vinum colchici in its cure.

Mrs. Daniels, æt. 32, Oct. 12th, 1819, was attacked with gout in the right ancle, foot, and great toe: the inflammation was intense, and the pain excessive. Six leeches were applied to the inflamed parts, and thirty minims of the vinum colchici, administered three times, carried off the pain, inflammation, and fever. She had had one attack two years before, which continued ten weeks; and, as yet, no return has occurred: consequently, the frequency of attack has not been increased, and the chronic gout was prevented.

Mr. R-b-ts used to be confined to his house for weeks, from his attacks of gout; he has employed colchicum, during four or five years, for the prompt cure of the paroxysm, and has neither experienced a greater frequency of return, nor has it assumed the chronic form.

An eminent judicial character desired my advice, on the 17th of November last. He had been attacked, two years and a half ago, with an acute paroxysm of gout, which merged into the chronic form, and finally left him with permanent œdema of the right extremity, and a thickening of the tendons, and tendinous expansions of the foot; which had not subsided when the present invasion took place, seven weeks ago. The chronic inflammation and pain now prevail in the right knee, foot, and great and little toes; the whole leg is œdematous; and the ligaments and bursæ mucosæ of the knee and ancle joints, and the tendons and tendinous expansions, with the sheaths of tendons about the ancle and foot, are thickened and swelled. The chronic pain and inflammation entirely yielded to six doses of vin. colch. m. xxv. repeated every night; and enabled us to employ bandage, and friction, first without, and afterwards with lin. camph. Under this treatment, the œdema and thickening daily and rapidly subsided for a fortnight, when an accident prevented a perseverance in these remedies for two days, and the œdema returned, with some slight pain, on the side of the foot; which my patient apprehended to be a return of gout, and proposed to take a little more colchicum. This I objected to; and, on resuming the use of bandage and friction, the pain and œdema soon subsided; and, by persevering in the use of friction and adhesive straps, I confidently hope to remove the thickened state of the ligaments, tendons, and tendinous expansion, that has continued so long. In this case, the chronic form was soon cured. It also teaches us, that gout may threaten to return when cured by colchicum, if an unremitting attention be not paid to the removal of the consequences of chronic gout; and that, by possibility, this circumstance may account for some of its reputed failures.

Without, however, multiplying cases to prove the facts I have dwelt upon, I will refer to those, of which I have a connected history, published in the Journal for November, 1821; and which were cured by small doses of vin. colchici, repeated for a few nights.

Case 1.—Mr. Sandison had no chronic disease, and has had but one paroxysm since April 4th, 1818.

Case 2.—Miss Davis has had no return, nor chronic gout, since October 17th, 1818.

Case 3.—Mrs. Jackson continues subject to gout twice a-year, and is very readily cured by vin. colchici, without the chronic form supervening, or an increased frequency of return.

Case 4.—J. Collins has had but one attack since June 12th, 1818.

Cases 6 and 7.—Had not chronic gout, and have not had any return.

Case 10.—Mr. H. Fisher has had one attack of gout since

May 2d, 1818; which was as readily cured, without assuming the chronic form, or a frequency of recurrence being induced.

Case 9.—W. Bunker. It has been already stated of this patient, that he was afflicted with chronic gout half the year, before I adopted the cure by vin. colchici; and that chalkstones successively formed on several of his joints. Since he has been my patient, from 1817, the gout, having been always cured in the early part of the acute stage, never assumed the chronic disease, nor formed chalkstones, nor has the frequency of the attacks increased.

In Case 11, of the Hon. V. G., the chronic disease was cured by a few doses of vin. colch. ʒss,; and enabled me to employ, with entire success, the means of curing œdema, thickened tendinous sheaths and expansions, &c. &c.

It appears to me, from the following passage in Dr. S.'s reply to my observations, that he concedes a specific action to colchicum, and retracts his former opinions; for, he says, "I may surely be allowed to prefer one preparation of colchicum to other preparations, and to employ the medicine upon my own principles, which differ from those which are acted upon by the practitioner, who not only uses the more active forms of colchicum, but administers the medicine with a view to obtain its immediate, *and more of its specific, action.*" If, in this passage, Dr. S. does not intend to concede a specific action to the colchicum, I repeat, let him discontinue the use of it in his treatment of gout; and it will prove as opprobrious and inefficient, as the *modus medendi* by colchicum is prompt and effectual.

Dr. S. points to "a general and comprehensive plan of treatment" of the acute stage alone, which occupies 158 pages of closely-printed octavo in detailing, in preference to one of extreme simplicity; but I am impressed with a belief, that some parts of it are not perfect, and others are objectionable. Thus, general bleeding is discussed, but local bleeding is deemed unnecessary and injurious, although, we think, we have derived considerable benefit from it in some cases. Emetics and mercurials I have not employed. Colchicum generally succeeds without producing any sensible diuretic, purgative, or sudorific effect: I, therefore, consider their excitement not absolutely necessary to the cure o gout, although purgatives are frequently useful in cases of pain in the bowels or constipation. The twenty-five pages on "pretended specifics" may be entirely omitted in the next edition; and the fifteen pages on the choice and use of narcotics will lose much of their importance, if the experience of others should be in consonance with my own; for I have so invariably found colchicum to be the best narcotic in gout, the soother of gouty pain, and imperceptible promoter of sleep, that I have never combined it with opium, but in one case, in which I accompanied its use with the addition of tr. opii m. x. It would be almost idle to consume the time of the reader in taking even a cursory view of the twenty pages Dr. S. has devoted to local applications: we cannot, however, consistently avoid entering a caveat against the use of the favourite "evaporating lotion." Dr. Kinglake has been most severely criticised for recommending the application of cold water; yet there is considerable resemblance between a cold and an evaporating lotion, as will appear from the following syllogism:—Cold applications in gout are frequently injurious; evaporation produces intense cold; therefore, evaporating lotions must be injurious. We have been informed so on good authority; and the profession will, no doubt, furnish instances of the bad effects of the "evaporating lotion," in producing retrocedent gout.

There is still a statement, in Dr. Scudamore's reply, to which it is my imperative duty to advert, as it might lead to a fatal mistake. I am there represented to state, that the quantity of one ounce of acet. colchici is *equivalent to 53⅓ grains in substance.* The fact is, that, after the perusal of Mr. Haden's Observations on Colchicum, I cautiously abstained from using the words "equivalent to grains in substance," lest some tyro, not in possession of the vin. or acet. colchici, should employ so large, and perhaps fatal, a quantity as a substitute; and I have, for this reason, every where adopted Dr. S.'s own words and term, "active properties;" and have observed, that Dr. S., in one ounce of acetum colchici, administers "all the active properties" of 53⅓ grains of colchicum; because he had previously assured us, in his treatise, that the acetum does take up "all the active properties" of the colchicum macerated in it; and, by courteous concession, *I have admitted the wine to do so too,* (although it be not the case,) that a comparison might be the more fairly drawn between them.

I will not disturb the satisfaction Dr. Scudamore's mind may derive from his experiments on dogs; for we are yet to learn that these animals are subject to gout, and because I conceive all that was necessary was long since ascertained by Sir E. Home's experiments, conducted by the late lamented Mr. R. Gatcombe; the effects of which I witnessed, and the results of which were published in the Transactions of the Royal Society.

Dr. S. concludes by declining "any future discussion of a controversial nature." Our sword has long rusted in the scabbard; and, as the same witty writer, who has so often reminded us that "de factis nil disputandum," tells us, "circumstances that must explain themselves are not worth a conjecture," we feel assured that, if Dr. S. will not oblige the public by informing it how it has occurred that his mode of exhibiting the vinum and tinctura colchici has so completely failed, the profession will, by experiment, ascertain it. I shall pursue the subject, and hope soon to have some new facts to lay before the public.

*37, Bedford-street, Covent Garden; Jan. 2, 1822.*

